# Acetylcholinesterase Inhibition of Diversely Functionalized Quinolinones for Alzheimer’s Disease Therapy

**DOI:** 10.3390/ijms21113913

**Published:** 2020-05-30

**Authors:** Óscar M. Bautista-Aguilera, Lhassane Ismaili, Mourad Chioua, Rudolf Andrys, Monika Schmidt, Petr Bzonek, María Ángeles Martínez-Grau, Christopher D. Beadle, Tatiana Vetman, Francisco López-Muñoz, Isabel Iriepa, Bernard Refouvelet, Kamil Musilek, José Marco-Contelles

**Affiliations:** 1 Department of Organic Chemistry and Inorganic Chemistry, Alcalá University, Alcalá de Henares, 28805 Madrid, Spain; oscar.bautista@uah.es (Ó.M.B.-A.); isabel.iriepa@uah.es (I.I.); 2 Laboratoire de Chimie Organique et Thérapeutique, Neurosciences Intégratives et Cliniques EA 481, University Bourgogne Franche-Comté, UFR Santé, 19, rue Ambroise Paré, F-25000 Besançon, France; lhassane.ismaili@univ-fcomte.fr (L.I.); bernard.refouvelet@univ-fcomte.fr (B.R.); 3 Laboratory of Medicinal Chemistry (IQOG, CSIC), C/ Juan de la Cierva 3, 28006 Madrid, Spain; mchioua@gmail.com; 4 Department of Chemistry, Faculty of Science, University of Hradec Kralove, Rokitanskeho 62, 500 03 Hradec Kralove, Czech Republic; rudolf.andrys@uhk.cz (R.A.); monika.schmidt@uhk.cz (M.S.); bzonepe@gmail.com (P.B.); 5 Lilly Research Laboratories, Eli Lilly & Company, Avda. de la Industria 30, Alcobendas, 28108 Madrid, Spain; mamgrau@gmail.com; 6 Lilly Research Centre, Eli Lilly & Company, Erl Wood Manor, Windlesham, Surrey GU20 6PH, UK; topher.beadle@gmail.com; 7 Lilly Research Laboratories, Eli Lilly & Company, Indianapolis, IN 46285, USA; vetman_tatiana@lilly.com; 8 Faculty of Health, Camilo José Cela University of Madrid (UCJC), 28692 Villafranca del Castillo, Spain; flopez@ucjc.edu; 9 Neuropsychopharmacology Unit, “Hospital 12 de Octubre” Research Institute, 28041 Madrid, Spain; 10 Institute of Chemical Research Andrés M. del Río, Alcalá University, Alcalá de Henares, 28805 Madrid, Spain

**Keywords:** Alzheimer’s disease, ChE/MAO inhibition, contilisant, dihydroquinolinones, docking, quinolinones, synthesis

## Abstract

In this communication, we report the synthesis and cholinesterase (ChE)/monoamine oxidase (MAO) inhibition of 19 quinolinones (**QN1**-**19**) and 13 dihydroquinolinones (**DQN1**-**13**) designed as potential multitarget small molecules (MSM) for Alzheimer’s disease therapy. Contrary to our expectations, none of them showed significant *human recombinant* MAO inhibition, but compounds **QN8**, **QN9**, and **DQN7** displayed promising *human recombinant* acetylcholinesterase (*hr*AChE) and butyrylcholinesterase (*hr*BuChE) inhibition. In particular, molecule **QN8** was found to be a potent and quite selective non-competitive inhibitor of *hr*AChE (IC_50_ = 0.29 µM), with K_i_ value in nanomolar range (79 nM). Pertinent docking analysis confirmed this result, suggesting that this ligand is an interesting hit for further investigation.

## 1. Introduction

Alzheimer’s disease (AD), a pathology of ageing affecting mainly the elderly [1], is a multifactorial, neurodegenerative disease characterized by a progressive decline of memory and cognitive faculties [2]. Although the aetiology of AD is unknown, oxidative stress, deficit of neurotransmitters, and neuronal death seem to play a critical role [3]. To date, all the efforts directed to find the origin of the disease have failed [4,5], but as the number of suffering people is dramatically increasing, there is a strong and urgent social need for therapeutic solutions. Acetylcholinesterase inhibitors (AChEI) such as donepezil, rivastigmine, and galantamine, able to increase the level of biogenic amines in the brain, or memantine, a non-competitive antagonist of the *N*-Methyl-*D*-Aspartate (NMDA) receptor, are the only drugs currently administered to AD patients [6]. In spite of their limitations, we think that the use of AChEI should be the starting point for designing more efficient clinical candidates for AD therapy. To do this, the concept and application of multitarget small molecules (MSM) [7] design strategy seem to us the best way to cope with it. The ability of MSM to simultaneously modulate receptors or inhibit the enzymatic systems involved in the progress of AD are the key factors of this approach [8,9]. However, the proof that this approach is possible for complex diseases such as AD still has to be confirmed [10].

**Figure 1:**
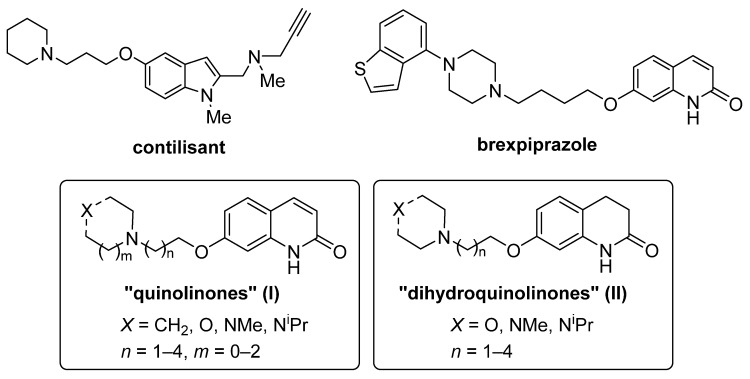
Structure of contilisant, brexpiprazole, “quinolinones” (I), and “dihydroquinolinones” (II), the new MSM designed in this work.

In a recent communication [11] we expanded the pharmacological profile of contilisant (Figure 1), our most advanced lead compound for AD. We showed that contilisant is an antioxidant, permeable, strong neuroprotective agent, able to increase the level of acetylcholine by inhibiting the cholinesterases (ChE) and monoamine oxidases (MAO), and modulating very specifically the histamine-3 (H3R) and sigma-1 (S1R) receptors. In addition, contilisant overcomes the efficiency of donepezil in the in vivo tests on suitable AD animal models [11].

However, in our current research project, one of the main concerns was the irreversible MAO inhibition shown by contilisant (Figure 1). Although this could be a point of debate [12], we decided to design new ligands behaving pharmacologically like contilisant, but acting as MAO-reversible inhibitors. In order to do this, we planned to substitute the *N*-methyl propargyl MAO-irreversible inhibition motif by a typical MAO-reversible pharmacophore, using it at the same time as the heterocyclic core instead of the indole ring. A number of options were possible to achieve these goals, as coumarins [13], chromones [14], and chalcones [15] are well known reversible and selective MAO-B inhibitors.

Thus, this simple design and recent communication on related chromenones [16] led us to select “quinolinones” (**I**, **QN**) and “dihydroquinolinones” (**II**, **DQN**) (Figure 1) as the MSM of choice, on the basis of the availability of the starting materials, as well as the simple synthetic schemes to synthesize them. Quinolinones have been previously described, with the antipsychotic drug “brexpiprazole” [17] (Figure 1) being one of the best known examples. Dihydroquinolinones (Figure 1) bearing different terminal amines have been reported as S1R antagonists for the potential use as analgesics [18], and as new MSM showing ChE/MAO inhibition [19,20] and histamine 3 receptor antagonism [21] for the treatment of AD.

**Figure 2:**
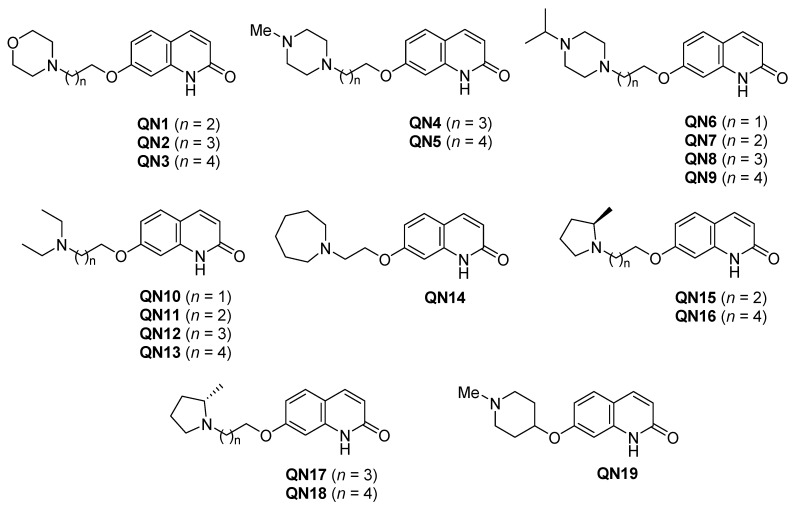
Structures of the “quinolinones” (**QN1**-**19**) described in this work.

In this work we describe the synthesis and biological evaluation of all these compounds designed as MSM for AD therapy, an effort that has allowed us to identify compound **QN8** (Figure 2) as a potent, selective and non-competitive *human recombinant* acetylcholinesterase (*hr*AChE) inhibitor.

## 2. Results and Discussion

### 2.1. Chemistry

**Figure 3:**
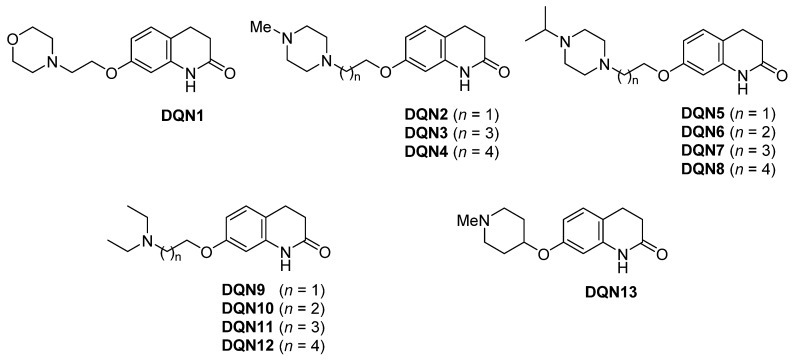
Structures of the “dihydroquinolinones” (**DQN1**-**13**) described in this work.

Prompted by our experience in the area, we submitted this project to the ASL (Automated Synthesis Lab) as part of the Eli Lilly’s Open Innovation Drug Discovery program [22], as this was an opportunity to synthesize compounds remotely, explore novel synthetic approaches, improve reaction efficiency, and test the feasibility of automatic synthetic processes to maximize the yield of targeted compounds. The proposal was accepted, and we started the remote synthesis of the designed ligands (Figure 2 and Figure 3).

In this structure–activity relationship (SAR) analysis we had the opportunity to evaluate the biological activity of different amines at variable distances from the quinolinone and dihydroquinolinone cores. In Figure 2, we show the structures of the “quinolinones” **QN1-19** that we prepared under Lilly’s ASL program. These 19 ligands contain a basic amine (morpholine, *N*-methylpiperazine, *N*-isopropylpiperazine, diethylamine, azepine, (*R*)-2-methylpyrrolidine, (*S*)-2-methylpyrrolidine, or *N*-methylpiperidine) linked at the quinolinone core by an alkoxy group of different sizes. Note that compounds **QN7** and **QN15-18** were prepared as formate salts. All these compounds are new (see the Materials and Methods section).

Similarly, in Figure 3, we show the structures of the “dihydroquinolinones” **DQN1-13** that we synthesized remotely at Lilly’s ASL. These 13 compounds bear morpholine, *N*-methylpiperazine, *N*-isopropylpiperazine, diethylamine, or *N*-methylpiperidine as the amino motif, with the corresponding linkers to the 3,4-dihydroquinolin-2(1*H*)-one core (see the Materials and Methods section). Most of the compounds in Figure 3 are new, but the morpholine **DQN1** [18], the *N*-isopropylpiperazines **DQN6-8** [19], and the diethylamines **DQN9** [21] and **DQN10-12** [19] have been previously described in the literature.

**Scheme 1:**
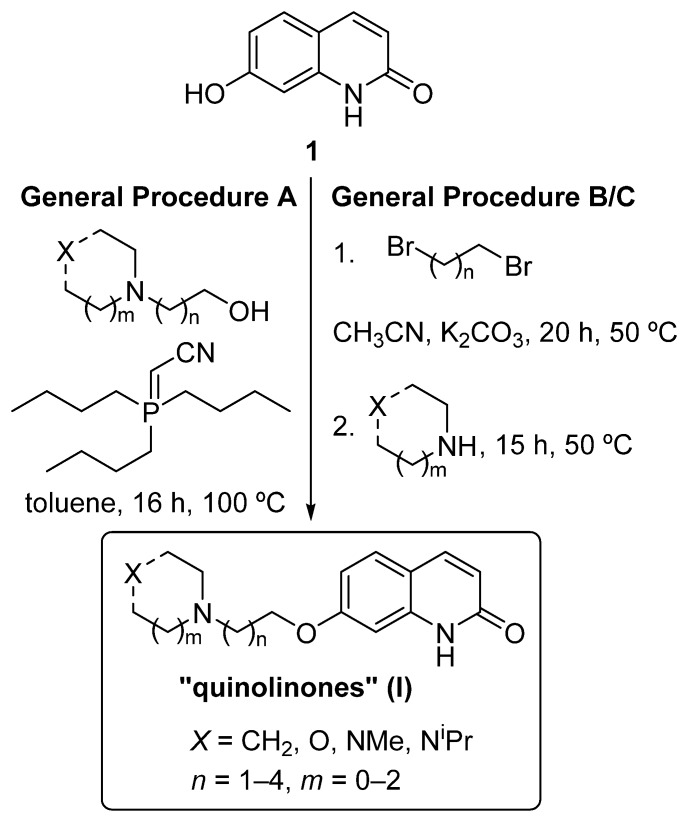
Synthetic methods for the preparation of the “quinolinones” (**QN1**-**19**).

**Scheme 2:**
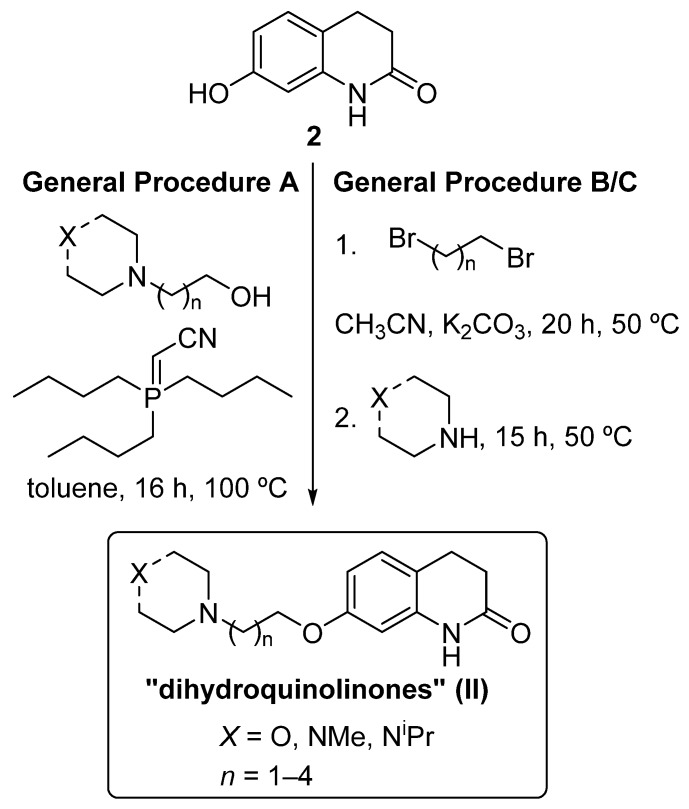
Synthetic methods for the preparation of the “dihydroquinolinones” (**DQN1**-**13**).

A number of compounds in these families were prepared by Mitsunobu reaction of commercially available 7-hydroxyquinolin-2(1*H*)-one (**1**) (Scheme 1) and 7-hydroxy-3,4-dihydroquinolin-2(1*H*)-one (**2**) (Scheme 2) with the appropriate alcohol. Another set of compounds were synthesized by *O*-alkylation of compounds **1** and **2** with the corresponding halides in two steps (Scheme 1 and Scheme 2). The present design is very versatile, allowing us also to change the length of the linker connecting the heterocyclic core with the cyclic (or acyclic) amine.

As described in the Materials and Methods section, starting from commercial 7-hydroxyquinolin-2(1*H*)-one (**1**) and the appropriate aminoalcohol, compounds **QN6** (10%), **QN10** (60%), **QN11** (60%), **QN14** (30%), and **QN19** (30%) (Figure 2) were obtained by Mitsunobu reaction using cyanomethylenetributylphosphorane, in toluene at reflux (Scheme 1) [23]. Compounds **QN1** (20%), **QN2** (20%), **QN3** (10%), **QN4** (20%), **QN5** (20%), **QN7** (30%), **QN8** (30%), **QN9** (10%), **QN12** (20%), **QN13** (50%), **QN15** (10%), **QN16** (10%), **QN17** (30%), and **QN18** (10%) (Figure 2) were synthesized by reacting K_2_CO_3_, 7-hydroxyquinolin-2(1*H*)-one (**1**), and the corresponding 1,*n*-dibromoalkane in CH_3_CN at 50 °C, with the subsequent addition of the appropriate amine (Scheme 1) [24].

Starting from commercially available 7-hydroxy-3,4-dihydroquinolin-2(1*H*)-one (**2**) and the appropriate aminoalcohol, compounds **DQN1** (80%), **DQN2** (80%), **DQN5** (30%), **DQN9** (70%), and **DQN13** (70%) (Figure 3) were obtained by Mitsunobu reaction using cyanomethylenetributylphosphorane in toluene at reflux (Scheme 2) [23]. Molecules **DQN3** (30%), **DQN4** (30%), **DQN6** (20%), **DQN7** (30%), **DQN8** (30%), **DQN10** (50%), **DQN11** (60%), and **DQN12** (50%) (Figure 3) were synthesized by reacting K_2_CO_3_, 7-hydroxy-3,4-dihydroquinolin-2(1*H*)-one (**2**), and the corresponding 1,*n*-dibromoalkane in CH_3_CN at 50 °C, with the subsequent addition of the appropriate amine (Scheme 2) [24].

All compounds showed proper analytical and spectroscopic data in good agreement with their structures.

### 2.2. Biological Evaluation

The compounds were screened for inhibition at *human recombinant* monoamine oxidases (*hr*MAO-A and *hr*MAO-B), *human recombinant* acetylcholinesterase (*hr*AChE) and *human recombinant* butyrylcholinesterase (*hr*BuChE), at two concentrations (1 and 10 µM). Clorgylin and pargylin were the standards used for *hr*MAO-A and *hr*MAO-B inhibition, respectively, at low concentrations (0.05 and 0.5 µM) due to their high ability to decrease MAO activity. Tacrine (1 and 10 µM) was chosen as the standard for *hr*AChE and *hr*BuChE inhibition.

The inhibition of *hr*MAO-A/B by novel compounds was rather weak compared with the standards used (data not shown).

These compounds were then tested on *hr*AChE/*hr*BuChE [25,26]. As a result, compounds **QN8**, **QN9**, and **DQN7** showed high *hr*AChE inhibition (88–95% at 10 µM concentration) and moderate *hr*BuChE inhibition (45–65% at 10 µM concentration).

**Figure 4:**
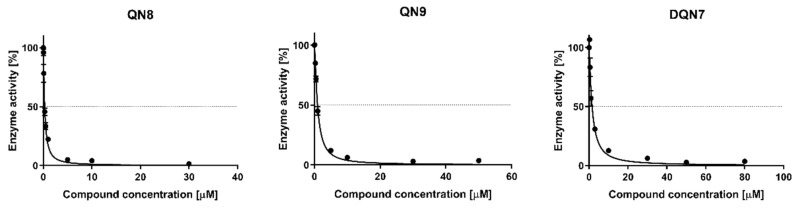
IC_50_ determination for *hr*AChE inhibition by **QN8**, **QN9**, and **DQN7**.

**Figure 5:**
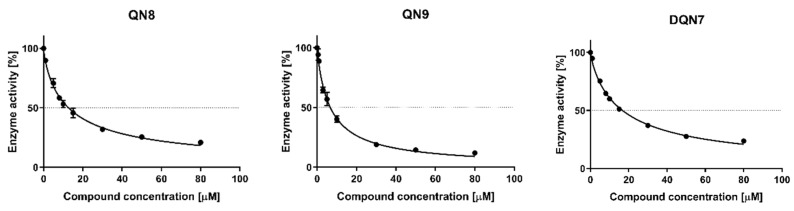
IC_50_ determination for *hr*BuChE inhibition by **QN8**, **QN9**, and **DQN7**.

**Figure 6:**
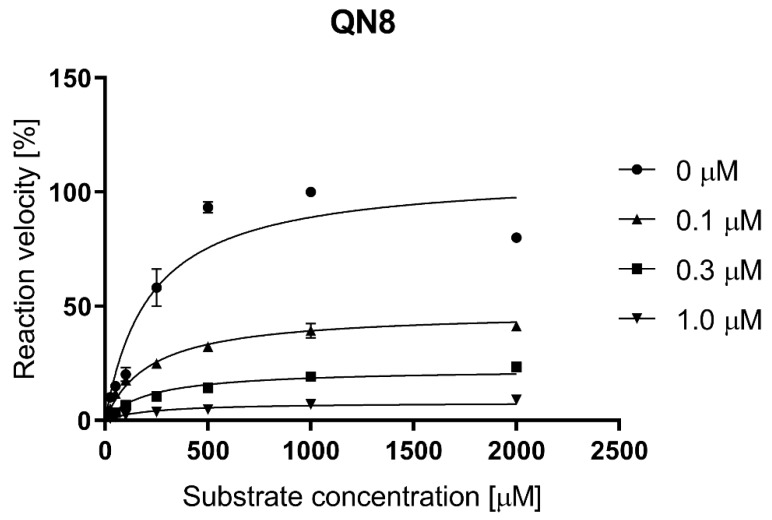
Inhibition kinetics of *hr*AChE by compound **QN8**.

On the basis of these data, three compounds (**QN8**, **QN9**, and **DQN7**) were chosen for IC_50_ determination in *hr*AChE and *hr*BuChE (Table 1, Figure 4 and Figure 5). Compound **QN8** demonstrated the highest inhibition for *hr*AChE (IC_50_ = 0.29 ± 0.02 µM) with the highest selectivity for *hr*AChE (*hr*BuChE IC_50_ = 12.73 ± 0.45 µM), and was further tested in kinetic experiments (Figure 6) [27]. Compound **QN8** resulted as a non-competitive inhibitor of *hr*AChE, with K_i_ value in nanomolar range (79 ± 7 nM). In comparison with donepezil, **QN8** is six-fold less potent as *hr*AChEI (Table 1), and shows a Ki value two-fold higher than donepezil (Ki = 39 nM) [28].

Compounds **DQN6-8** and **DQN10-12** have been previously described and tested as MAO and ChE inhibitors [19]. We observed low *hr*MAO inhibition at 1 and 10 µM concentration (data not shown) and did not determine their IC_50_. In a formerly published study [19], *hr*MAO inhibition was reported in the double digit micromolar range for most of the compounds, but the source of MAOs was not mentioned or referenced.

Regarding the inhibition of the *h*ChEs, the previous data [19] were different from ours, but used eel, equine, or rat AChE and BuChE enzymes. Because of the higher relevance, we think that only data determined for human enzymes should be considered for further investigation.

Our SAR design was based on two similar cores, a variable linker and a limited number of differently functionalized basic centres (Figure 2 and Figure 3). On the basis of the comparison between compounds **QN8** (0.29 µM) and **DQN7** (1.58 µM), the quinolinone core looked more potent than the semi-unsaturated dihydroquinolinone as *hr*AChE inhibitor. The isopropylpiperazine seemed also essential for the *hr*AChE activity with the butoxy group as the optimal linker between the core and basic centre. The branched isopropyl substituent boosted the *hr*AChE inhibition as observed when comparing **QN8** (0.29 µM) with the methylpiperazine **QN4** (low inhibition percentage). This assessment is also valid for the compounds **QN9** (0.96 µM) and **DQN7** (1.58 µM) vs. **QN5** and **DQN3** (low inhibition percentage). These SAR findings open new directions to develop further modified *hr*AChE inhibitors with *hr*BuChE selectivity.

### 2.3. Computational Chemistry: Docking of Compound QN8

To justify the observed in vitro enzymatic activity and shed light into the *hr*AChE active site and binding mode of compound **QN8**, we performed molecular docking studies using software AutoDock Vina 1.1.2 [29].

**Figure 7:**
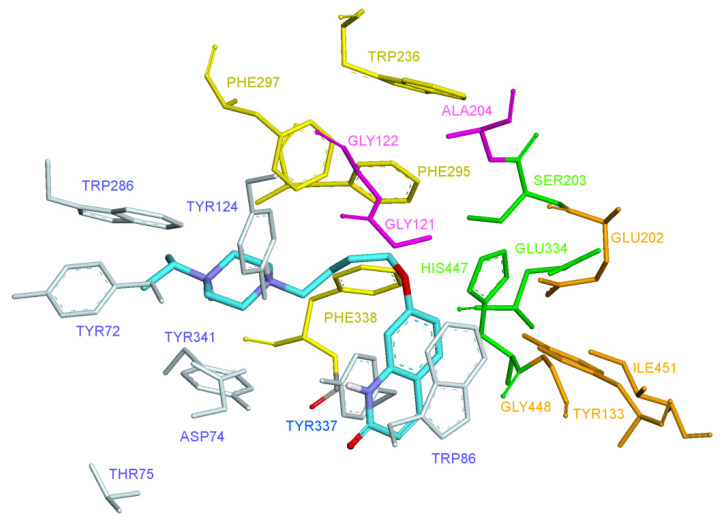
Binding mode of **QN8** in the active site of *hr*AChE. Compound **QN8** is rendered as sticks (carbon atoms in blue) and the side chain conformations of the mobile residues are illustrated in light blue. Catalytic triad (CT) is coloured in green, oxyanion hole (OH) in magenta, anionic subsite (AS) in orange except Trp86, the acyl binding pocket (ABP) in yellow, and peripheral anionic site (PAS) in light blue.

**Figure 8:**
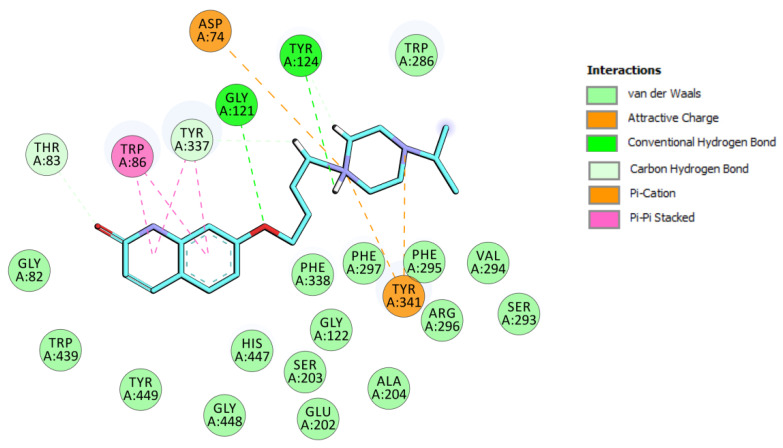
2D schematic view of the interactions between **QN8** and *hr*AChE.

Compound **QN8** showed quite high binding affinity (−10.3 kcal/mol), indicating a tight binding to the enzyme. The docking analysis revealed that compound **QN8** spanned the narrow AChE active site interacting with the anionic subsite (AS) halfway down the gorge, and in the mouth of the gorge at the peripheral anionic site (PAS), but not with the catalytic triad (CT: residues His447, Glu334, and Ser203) of *h*AChE (Figure 7). In this binding mode, the quinolinone ring interacted with Trp86 (at the AS) and with Tyr337 via π–π stacking interactions. The diprotonated piperazine moiety interacted with Asp74 and Tyr341, at the PAS, via attractive charge and π-cation interactions, respectively. The oxygen ether formed a hydrogen bond with Gly121 in the oxyanion hole (OH), while Tyr124 formed a hydrogen bond with the hydrogen of one of the quaternized nitrogen of the piperazine moiety. In addition, some carbon hydrogen interactions were observed between the ligand and Thr83, Tyr124, and Ty337 (Figure 8).

In conclusion, docking results based on the interactions of compound **QN8** at the AS, PAS and OH are in good agreement with the experimental kinetic data, indicating and confirming a non-competitive type of inhibition.

### 2.4. Predicted Physico-Chemical Properties Analysis for Ligand QN8

The physico-chemical properties of ligand **QN8** were evaluated with QikProp module of Schrodinger (QikProp, version 5.1, Schrodinger, LLC, New York, NY, 2017-1). The selected properties are known to influence metabolism, cell permeation, and bioavailability (Table 2).

Central Nervous System (CNS) drugs tend to be more lipophilic, less polar, less flexible, and have lower molecular weight and molecular volume than drugs used for other therapeutics. Usually, CNS drugs show values of molecular weight (MW) < 450, number of hydrogen bond donors < 3, number of hydrogen bond acceptors < 7, partition coefficient (QPlogPo/w) < 5, polar surface area (PSA) < 90 square Å, number of rotatable bonds < 8, and hydrogen bonds < 8.

The partition coefficient (QPlogPo/w), which is critical for estimation of absorption within the body, is 2.449 for ligand **QN8**. A value that ranges 1 to 3 is most favourable for blood–brain barrier (BBB) penetration and CNS activity. The gut–blood barrier permeability was predicted using Caco-2 cell permeability (QPPCaco) as model and it showed good value at 87.998 (<25 poor, >500 great). Further, the prediction for human serum albumin binding using QPlogKhsa showed that the value for **QN8** lay within the expected range (from −1.5 to 1.5). Likewise, the brain/blood partition coefficient (QPlogBB) showed a satisfactory value (Table 2). The percentage of oral drug absorption predicted for the test compound was adequate, with a high percentage (>70%) of human oral absorption, indicating their possibilities in oral drug formulation.

The predicted properties of compound **QN8** (Table 2) were in the ranges defined by QikProp for 95% of known oral drugs and also satisfied the Lipinski’s rule of five [30]. In conclusion, on the basis of the physico-chemical properties shown in Table 2, compound **QN8** shows characteristics that are typical of a drug-like molecule with potential BBB permeability.

## 3. Materials and Methods

### 3.1. Chemistry

#### 3.1.1. General Methods

^1^H-NMR (400 MHz) spectra were recorded on a Bruker Avance III 400 NMR spectrometer (Bruker, Billerica, MA, USA). Chemical shifts δ are given in parts per million referring to the signal centre using the solvent peaks for reference: DMSO-*d*_6_ 2.49 ppm. LC/MS were recorded in an Agilent HP1100 liquid chromatography system (Agilent Technologies, Santa Clara, CA, USA). Electrospray mass spectrometry measurements (acquired in positive mode) were performed on a mass selective detector quadrupole spectrometer interfaced to the HP1100 HPLC. The conditions followed were as follows: column: HPH Phenomenex Kinetix EVO 2.6u, 2.1 × 30 mm; flow rate: 0.85 mL/min; gradient: 5–100% B; run time: 2 min; solvent A: 10 mM ammonium bicarbonate (pH = 10); solvent B: acetonitrile. The purity of all products, as checked with LC/MS using a diode array detector coupled to a mass spectrometer, was higher than 95%.

#### 3.1.2. General Procedures for the Synthesis of Quinolinones (QNs)

(a) General Procedure A via Mitsunobu reaction: A mixture of 7-hydroxyquinolin-2(1*H*)-one (0.5 mmol), the appropriate aminoalcohol (0.5 mmol), and cyanomethylenetributylphosphorane (0.7 mmol) in toluene (5 mL) was heated at 100 °C for 16 h. The reaction mixture was diluted with methanol and loaded into 10 g Strong Cation Exchange (SCX) column. Resin was washed with methanol, and crude product was eluted in 2N ammonia in methanol before evaporation of the solvent. The residue was purified by column chromatography (CH_2_Cl_2_/MeOH (from 1% to 5%)).

(b) General Procedure B via *O*-Alkylation: Step 1. A mixture of potassium carbonate (2.5 mmol), 7-hydroxyquinolin-2(1*H*)-one (0.5 mmol), and the corresponding 1,*n*-dibromoalkane (1.0 mmol) in acetonitrile (5 mL) was heated at 50 °C for 20 h. After cooling, acetonitrile (10 mL) was added, the inorganic salts were filtered off, and the solvent was evaporated. Step 2. To a stirred solution of the crude intermediate in acetonitrile (5 mL), we added the appropriate amine (2.0 mmol). After stirring at 50 °C for 15 h, we diluted the reaction mixture with methanol and loaded it into 10 g SCX resin. Resin was washed with methanol, and crude product was eluted with 2N ammonia in methanol before evaporation of the solvent. The residue was purified by column chromatography (CH_2_Cl_2_/MeOH (from 1% to 5%)).

(c) General Procedure C via *O*-Alkylation: Step 1. A mixture of potassium carbonate (2.5 mmol), 7-hydroxyquinolin-2(1*H*)-one (0.5 mmol), and the corresponding 1,*n*-dibromoalkane (1.0 mmol) in acetonitrile (5 mL) was heated at 50 °C for 20 h. After cooling, acetonitrile (10 mL) was added, the inorganic salts were filtered off, and the solvent was evaporated. Step 2. To a stirred solution of the crude intermediate in acetonitrile (5 mL), we added the appropriate amine (0.5 mmol). After stirring at 50 °C for 15 h, we diluted the reaction mixture with methanol and loaded it into 10 g SCX resin. Resin was washed with methanol, and crude product was eluted with 2N ammonia in methanol before evaporation of the solvent. The residue was purified by column chromatography (CH_2_Cl_2_/MeOH (from 1% to 5%)).

*7-(3-Morpholinopropoxy)quinolin-2(1H)-one* (**QN1**). Following General Procedure B, the reaction of a mixture of potassium carbonate (345.5 mg, 2.5 mmol), 7-hydroxyquinolin-2(1*H*)-one (80.6 mg, 0.5 mmol), and 1,3-dibromopropane (202 mg, 1.0 mmol) in acetonitrile (5 mL) afforded a crude intermediate that was suspended in acetonitrile (5 mL), and treated with morpholine (174.3 mg, 2.0 mmol) to give compound **QN1** (22.73 mg, 20% yield). ^1^H-NMR (400 MHz, DMSO-*d*_6_) δ: 11.55 (br s, 1H), 7.81 (d, *J* = 9.5 Hz, 1H), 7.59 (dd, *J* = 8.3, 1.3 Hz, 1H), 6.79–6.75 (m, 2H), 6.30 (d, *J* = 9.5 Hz, 1H), 4.05 (t, *J* = 6.5 Hz, 2H), 3.58–3.54 (m, 4H), 2.40–2.37 (m, 6H), 1.91–1.86 (m, 2H). LC/MS *m*/*z* 289.2 (M + H), t_R_ = 0.65 min.

*7-(4-Morpholinobutoxy)quinolin-2(1H)-one* (**QN2**). Following General Procedure B, the reaction of a mixture of potassium carbonate (345.5 mg, 2.5 mmol), 7-hydroxyquinolin-2(1*H*)-one (80.6 mg, 0.5 mmol), and 1,4-dibromobutane (216 mg, 1.0 mmol) in acetonitrile (5 mL) afforded a crude intermediate that was suspended in acetonitrile (5 mL), and treated with morpholine (174.3 mg, 2.0 mmol) to give compound **QN2** (20.53 mg, 20% yield). ^1^H-NMR (400 MHz, DMSO-*d*_6_) δ: 11.55 (br s, 1H), 7.80 (d, *J* = 9.5 Hz, 1H), 7.55 (dd, *J* = 8.4, 1.3 Hz, 1H), 6.79–6.76 (m, 2H), 6.29 (d, *J* = 9.5 Hz, 1H), 4.03 (t, *J* = 6.4 Hz, 2H), 3.56-3.52 (m, 4H), 2.33–2.30 (m, 6H), 1.76–1.73 (m, 2H), 1.58–1.55 (m, 2H). LC/MS *m*/*z* 303.2 (M + H), t_R_ = 0.71 min.

*7-(5-Morpholinopentyloxy)quinolin-2(1H)-one***(QN3)**. Following General Procedure C, the reaction of a mixture of potassium carbonate (345.5 mg, 2.5 mmol, 7-hydroxyquinolin-2(1*H*)-one (80.6 mg, 0.5 mmol), and 1,5-dibromopentane (230 mg, 1.0 mmol) in acetonitrile (5 mL) afforded a crude intermediate that was suspended in acetonitrile (5 mL), and treated with morpholine (43.6 mg, 0.5 mmol) to give compound **QN3** (19.87 mg, 10% yield). ^1^H-NMR (400 MHz, DMSO-*d*_6_) δ: 11.56 (br s, 1H), 7.80 (d, *J* = 9.5 Hz, 1H), 7.55 (dd, *J* = 8.3, 1.3 Hz, 1H), 6.79–6.76 (m, 2H), 6.30 (d, *J* = 9.5 Hz, 1H), 4.01 (t, *J* = 6.5 Hz, 2H), 3.56–3.52 (m, 4H), 2.30–2.26 (m, 6H), 1.76–1.73 (m, 2H), 1.54–1.37 (m, 4H). LC/MS *m*/*z* 317.2 (M + H), t_R_ = 0.77 min.

*7-[4-(4-Methylpiperazin-1-yl)butoxy]quinolin-2(1H)-one***(QN4)**. Following General Procedure B, the reaction of a mixture of potassium carbonate (345.5 mg, 2.5 mmol), 7-hydroxyquinolin-2(1*H*)-one (80.6 mg, 0.5 mmol), and 1,4-dibromobutane (216 mg, 1.0 mmol) in acetonitrile (5 mL) afforded a crude intermediate that was suspended in acetonitrile (5 mL), and treated with *N*-methylpiperazine (200.3 mg, 2.0 mmol) to give compound **QN4** (29.38 mg, 20% yield). ^1^H-NMR (400 MHz, DMSO-*d*_6_) δ: 11.56 (br s, 1H), 7.80 (d, *J* = 9.5 Hz, 1H), 7.55 (dd, *J* = 8.4, 1.3 Hz, 1H), 6.83–6.76 (m, 2H), 6.29 (d, *J* = 9.5 Hz, 1H), 4.02 (t, *J* = 6.4 Hz, 2H), 2.37–2.27 (m, 7H), 2.14 (s, 3H), 1.75–1.73 (m, 2H), 1.57–1.55 (m, 2H) (the missing 3H must be hidden under the DMSO-d_6_ signal). LC/MS *m*/*z* 316.2 (M + H), t_R_ = 0.68 min.

*7-[5-(4-Methylpiperazin-1-yl)pentyloxy]quinolin-2(1H)-one***(QN5)**. Following General Procedure C, the reaction of a mixture of potassium carbonate (345.5 mg, 2.5 mmol), 7-hydroxyquinolin-2(1H)-one (80.6 mg, 0.5 mmol), and 1,5-dibromopentane (230 mg, 1.0 mmol) in acetonitrile (5 mL) afforded a crude intermediate that was suspended in acetonitrile (5 mL), and treated with *N*-methylpiperazine (50.08 mg, 0.5 mmol) to give compound **QN5** (27.16 mg, 20% yield). ^1^H-NMR (400 MHz, DMSO-*d*_6_) δ: 11.48 (br s, 1H), 7.80 (d, *J* = 9.4 Hz, 1H), 7.59 (dd, *J* = 8.4, 1.3 Hz, 1H), 6.78–6.76 (m, 2H), 6.29 (d, *J* = 9.4 Hz, 1H), 4.00 (t, *J* = 6.4 Hz, 2H), 2.32–2.26 (m, 10H), 2.14 (s, 3H), 1.75–1.72 (m, 2H), 1.44–1.41 (m, 4H). LC/MS *m*/*z* 330.2 (M + H), t_R_ = 0.73 min.

*7-[2-(4-Isopropylpiperazin-1-yl)ethoxy]quinolin-2(1H)-one***(QN6)**. Following General Procedure A, 7-hydroxyquinolin-2(1*H*)-one (80.6 mg, 0.5 mmol) and 2-(4-isopropylpiperazin-1-yl)ethan-1-ol (86.1 mg, 0.5 mmol) were reacted with cyanomethylenetributylphosphorane (169 mg, 0.7 mmol) in toluene (5 mL) to give compound **QN6** (20.91 mg, 10% yield). ^1^H-NMR (400 MHz, DMSO-*d*_6_) δ: 11.53 (br s, 1H), 7.81 (d, *J* = 9.4 Hz, 1H), 7.56 (dd, *J* = 8.4, 1.4 Hz, 1H), 6.82–6.79 (m, 2H), 6.30 (d, *J* = 9.4 Hz, 1H), 4.10 (t, *J* = 5.8 Hz, 2H), 2.70 (t, *J* = 5.8 Hz, 2H), 2.59–2.27 (m, 9H), 0.96 (d, *J* = 6.3 Hz, 6H). LC/MS *m*/*z* 316.2 (M + H), t_R_ = 0.69 min.

*7-[3-(4-Isopropylpiperazin-1-yl)propoxy]quinolin-2(1H)-one formate salt***(QN7)**. Following General Procedure B, the reaction of a mixture of potassium carbonate (345.5 mg, 2.5 mmol), 7-hydroxyquinolin-2(1H)-one (80.6 mg, 0.5 mmol), and 1,3-dibromopropane (202 mg, 1.0 mmol) in acetonitrile (5 mL) afforded a crude intermediate that was suspended in acetonitrile (5 mL), and treated with 1-isopropylpiperazine (256.4 mg, 2.0 mmol) to give compound **QN7** as free base. Treatment with 0.1% formic acid/H_2_O afforded compound **QN7** (46.98 mg, 30% yield). ^1^H-NMR (400 MHz, DMSO-*d*_6_) δ: 11.56 (s, 1H), 7.81 (d, *J* = 9.5 Hz, 1H), 7.55 (dd, *J* = 8.5, 1.3 Hz, 1H), 6.81–6.79 (m, 2H), 6.30 (d, *J* = 9.5 Hz, 1H), 4.06 (t, *J* = 5.9 Hz, 2H), 2.59–2.38 (m, 11H), 1.90–1.87 (m, 2H), 0.96 (d, *J* = 6.5 Hz, 6H). LC/MS *m*/*z* 330.2 (M + H), t_R_ = 0.27 min.

*7-[4-(4-Isopropylpiperazin-1-yl)butoxy]quinolin-2(1H)-one***(QN8)**. Following General Procedure C, the reaction of a mixture of potassium carbonate (345.5 mg, 2.5 mmol), 7-hydroxyquinolin-2(1H)-one (80.6 mg, 0.5 mmol), and 1,4-dibromobutane (216 mg, 1.0 mmol) in acetonitrile (5 mL) afforded a crude intermediate that was suspended in acetonitrile (5 mL), and treated with 1-isopropylpiperazine (64.1 mg, 0.5 mmol) to give compound **QN8** (49.56 mg, 30% yield). ^1^H-NMR (400 MHz, DMSO-*d*_6_) δ: 11.56 (br s, 1H), 7.80 (d, *J* = 9.5 Hz, 1H), 7.55 (dd, *J* = 8.3, 1.3 Hz, 1H), 6.79–6.77 (m, 2H), 6.29 (d, *J* = 9.5 Hz, 1H), 4.02 (t, *J* = 6.4 Hz, 2H), 2.58 (t, *J* = 6.5 Hz, 2H), 2.40–2.30 (m, 9H), 1.81–1.69 (m, 2H), 1.63–1.50 (m, 2H), 0.95 (d, *J* = 6.5 Hz, 6H). LC/MS *m*/*z* 344.2 (M + H), t_R_ = 0.80 min.

*7-[5-(4-Isopropylpiperazin-1-yl)pentyloxy]quinolin-2(1H)-one***(QN9)**. Following General Procedure C, the reaction of a mixture of potassium carbonate (345.5 mg, 2.5 mmol), 7-hydroxyquinolin-2(1H)-one (80.6 mg, 0.5 mmol), and 1,5-dibromopentane (230 mg, 1.0 mmol) in acetonitrile (5 mL) afforded a crude intermediate that was suspended in acetonitrile (5 mL) and treated with 1-isopropylpiperazine (64.1 mg, 0.5 mmol) to give compound **QN9** (22.19 mg, 10% yield). ^1^H-NMR (400 MHz, DMSO-*d*_6_) δ: 11.55 (br s, 1H), 7.80 (d, *J* = 9.5 Hz, 1H), 7.55 (dd, *J* = 8.3, 1,3 Hz, 1H), 6.80–6.77 (m, 2H), 6.29 (dd, *J* = 9.5 Hz, 1H), 4.00 (t, *J* = 6.4 Hz, 2H), 2.57-1.73 (m, 11H), 1.75–1.65 (m, 2H), 1.45-1.42 (m, 4H), 0.95 (d, *J* = 6.5 Hz, 6H). LC/MS *m*/*z* 358.2 (M + H), t_R_ = 0.86 min.

*7-[2-(Diethylamino)ethoxy]quinolin-2(1H)-one***(QN10)**. Following General Procedure A, 7-hydroxyquinolin-2(1*H*)-one (80.6 mg, 0.5 mmol) and 2-(diethylamino)ethan-1-ol (58.6 mg, 0.5 mmol) were reacted with cyanomethylenetributylphosphorane (169 mg, 0.7 mmol), in toluene (5 mL, 47.3 mmol) to give compound **QN10** (77.06 mg, 60% yield). ^1^H-NMR (400 MHz, DMSO-*d*_6_) δ: 11.56 (s, 1H), 7.81 (d, *J* = 9.4 Hz, 1H), 7.56 (dd, *J* = 8.4, 1.3 Hz, 1H), 6.79–6.77 (m, 2H), 6.30 (d, *J* = 9.4 Hz, 1H), 4.05 (t, *J* = 6.2 Hz, 2H), 2.80 (t, *J* = 6.2 Hz, 2H), 2.57 (q, *J* = 7.1 Hz, 4H), 0.99 (t, *J* = 7.1 Hz, 6H). LC/MS *m*/*z* 261.2 (M + H), t_R_ = 0.78 min.

*7-[3-(Diethylamino)propoxy]quinolin-2(1H)-one***(QN11)**. Following General Procedure A, 7-hydroxyquinolin-2(1*H*)-one (80.6 mg, 0.5 mmol) and 3-(diethylamino)propan-1-ol (65.6 mg, 0.5 mmol) were reacted with cyanomethylenetributylphosphorane (169 mg, 0.7 mmol) in toluene (5 mL) to give compound **QN11** (77.0 mg, 60% yield). ^1^H-NMR (400 MHz, DMSO-*d*_6_) δ: 11.51 (br s, 1H), 7.80 (d, *J* = 9.5 Hz, 1H), 7.55 (dd, *J* = 8.5, 1.3 Hz, 1H), 6.80–6.77 (m, 2H), 6.29 (d, *J* = 9.5 Hz, 1H), 4.04 (t, *J* = 6.3 Hz, 2H), 2.46 (q, *J* = 7.1 Hz, 4H), 1.84–1.81 (m, 2H), 0.95 (t, *J* = 7.1 Hz, 6H) (the missing 2H must be hidden under the DMSO-d_6_ signal). LC/MS *m*/*z* 275.2 (M + H), t_R_ = 0.81 min.

*7-[4-(Diethylamino)butoxy]quinolin-2(1H)-one***(QN12)**. Following General Procedure B, the reaction of a mixture of potassium carbonate (345.5 mg, 2.5 mmol), 7-hydroxyquinolin-2(1*H*)-one (80.6 mg, 0.5 mmol), and 1,4-dibromobutane (216 mg, 1.0 mmol) in acetonitrile (5 mL) afforded a crude intermediate that was suspended in acetonitrile (5 mL), and treated with diethylamine (146.3 mg, 2.0 mmol) to give compound **QN12** (27.81 mg, 20% yield). ^1^H-NMR (400 MHz, DMSO-*d*_6_) δ: 11.56 (s, 1H), 7.80 (d, *J* = 9.4 Hz, 1H), 7.56 (dd, *J* = 8.4, 1.3 Hz, 1H), 6.82–6.78 (m, 2H), 6.29 (d, *J* = 9.4 Hz, 1H), 4.02 (t, *J* = 6.6 Hz, 2H), 2.43 (q, *J* = 7.1 Hz, 4H), 1.77–1.71 (m, 2H), 1.57–1.51 (m, 2H), 0.95 (t, *J* = 7.1 Hz, 6H) (the missing 2H must be hidden under the DMSO-d_6_ signal). LC/MS *m*/*z* 289.2 (M + H), t_R_ = 0.84 min.

*7-[5-(Diethylamino)pentyloxy]quinolin-2(1H)-one***(QN13)**. Following General Procedure B, the reaction of a mixture of potassium carbonate (345.5 mg, 2.5 mmol), 7-hydroxyquinolin-2(1*H*)-one (80.6 mg, 0.5 mmol), and 1,5-dibromopentane (230 mg, 1.0 mmol) in acetonitrile (5 mL) afforded a crude intermediate that was suspended in acetonitrile (5 mL), and treated with diethylamine (146.3 mg, 2.0 mmol) to give compound **QN13** (82.72 mg, 50% yield). ^1^H-NMR (400 MHz, DMSO-*d*_6_) δ: 11.56 (br s, 1H), 7.80 (d, *J* = 9.5 Hz, 1H), 7.55 (dd, *J* = 8.3, 1.3 Hz, 1H), 6.80–6.77 (m, 2H), 6.29 (d, *J* = 9.5 Hz, 1H), 4.00 (t, *J* = 6.5 Hz, 2H), 2.49–2.35 (m, 6H), 1.79–1.73 (m, 2H), 1.46–1.38 (m, 4H), 0.94 (t, *J* = 7.1 Hz, 6H). LC/MS *m*/*z* 303.2 (M + H), t_R_ = 0.90 min.

*7-[2-(Azepan-1-yl)ethoxy]quinolin-2(1H)-one***(QN14)**. Following General Procedure A, 7-hydroxyquinolin-2(1*H*)-one (80.6 mg, 0.5 mmol) and 2-(azepan-1-yl)ethan-1-ol (71.6 mg, 0.5 mmol) were reacted with cyanomethylenetributylphosphorane (169 mg, 0.7 mmol) in toluene (5 mL, 47.3 mmol) to give compound **QN14** (46.52 mg, 30% yield). ^1^H-NMR (400 MHz, DMSO-*d*_6_) δ: 8.28 (d, *J* = 9.0 Hz, 1H), 7.62 (dd, *J* = 8.8, 0.7 Hz, 1H), 7.44–7.40 (m, 2H), 6.33 (d, *J* = 9.0 Hz, 1H), 4.19 (t, *J* = 5.7 Hz, 2H), 3.62–3.55 (m, 4H), 2.73 (t, *J* = 5.7 Hz, 2H), 2.49–2.43 (m, 8H). LC/MS *m*/*z* 287.2 (M + H), t_R_ = 0.90 min.

*(R)-7-[3-(2-Methylpyrrolidin-1-yl)propoxy]quinolin-2(1H)-oneformate salt***(QN15)**. Following General Procedure B, the reaction of a mixture of potassium carbonate (345.5 mg, 2.5 mmol), 7-hydroxyquinolin-2(1*H*)-one (80.6 mg, 0.5 mmol), and 1,3-dibromopropane (202 mg, 1.0 mmol) in acetonitrile (5 mL) afforded a crude intermediate that was suspended in acetonitrile (5 mL) and treated with (*R*)-2-methylpyrrolidine (170.3 mg, 2.0 mmol) to give compound **QN15** as a free base. Treatment with 0.1% formic acid/H_2_O afforded compound **QN15** (18.05 mg, 10% yield). ^1^H-NMR (400 MHz, DMSO-*d*_6_) δ: 11.55 (s, 1H), 7.80 (d, *J* = 9.4 Hz, 1H), 7.55 (dd, *J* = 8.4, 1.3 Hz, 1H), 6.86–6.75 (m, 2H), 6.29 (d, *J* = 9.4 Hz, 1H), 4.05 (t, *J* = 6.4 Hz, 2H), 3.10–3.06 (m, 1H), 2.92–2.89 (m, 1H), 2.26–2.23 (m, 1H), 2.15–2.10 (m, 1H), 2.05–2.00 (m, 1H), 1.93–1.84 (m, 3H), 1.73–1.58 (m, 2H), 1.35–1.21 (m, 1H), 1.00 (d, *J* = 6.0 Hz, 3H). LC/MS *m*/*z* 287.2 (M + H), t_R_ = 0.39 min.

*(R)-7-[5-(2-Methylpyrrolidin-1-yl)pentyloxy]quinolin-2(1H)-one formate salt***(QN16)**. Following General Procedure B, the reaction of a mixture of potassium carbonate (345.5 mg, 2.5 mmol), 7-hydroxyquinolin-2(1*H*)-one (80.6 mg, 0.5 mmol), and 1,5-dibromopentane (230 mg, 1.0 mmol) in acetonitrile (5 mL) afforded a crude intermediate that was suspended in acetonitrile (5 mL) and treated with (*R*)-2-methylpyrrolidine (170.3 mg, 2.0 mmol) to give compound **QN16** as free base. Treatment with 0.1% formic acid/H_2_O afforded compound **QN16** (31.92 mg, 10% yield). ^1^H-NMR (400 MHz, DMSO-*d*_6_) δ: 11.53 (br s, 1H), 7.80 (d, *J* = 9.5 Hz, 1H), 7.55 (dd, *J* = 8.4, 1.2 Hz, 1H), 6.80–6.75 (m, 2H), 6.29 (d, *J* = 9.5 Hz, 1H), 4.01 (t, *J* = 6.5 Hz, 2H), 3.05–3.01 (m, 1H), 2.74–2.65 (m, 1H), 2.21–2.16 (m, 1H), 1.99–1.94 (m, 2H), 1.86–1.71 (m, 3H), 1.64–1.57 (m, 2H), 1.55–1.36 (m, 4H), 1.30–1.23 (m, 1H), 1.00 (d, *J* = 6.0 Hz, 3H). LC/MS *m*/*z* 315.2 (M + H), t_R_ = 0.49 min.

*(S)-7-[4-(2-Methylpyrrolidin-1-yl)butoxy]quinolin-2(1H)-oneformate salt***(QN17)**. Following General Procedure B, the reaction of a mixture of potassium carbonate (345.5 mg, 2.5 mmol), 7-hydroxyquinolin-2(1*H*)-one (80.6 mg, 0.5 mmol), and 1,4-dibromobutane (216 mg, 1.0 mmol) in acetonitrile (5 mL) afforded a crude intermediate that was suspended in acetonitrile (5 mL), and treated with (*S*)-2-methylpyrrolidine (170.3 mg, 2.0 mmol) to give compound **QN17** as free base. Treatment with 0.1% formic acid/H_2_O afforded compound **QN17** (39.42 mg, 30% yield). ^1^H-NMR (400 MHz, DMSO-*d*_6_) δ: 11.57 (s, 1H), 8.18 (s, 1H), 7.80 (d, *J* = 9.5 Hz, 1H), 7.55 (dd, *J* = 8.3, 1.4 Hz, 1H), 6.80–6.78 (m, 2H), 6.29 (dd, *J* = 8.5, 1.8 Hz, 1H), 4.03 (t, *J* = 6.4 Hz, 2H), 3.17–3.08 (m, 1H), 2.84–2.78 (m, 1H), 2.32–2.28 (m, 1H), 2.20–2.06 (m, 2H), 1.92–1.57 (m, 7H), 1.34–1.25 (m, 1H), 1.05 (d, *J* = 6.1 Hz, 3H). LC/MS *m*/*z* 301.2 (M + H), t_R_ = 0.42 min.

*(S)-7-[5-(2-Methylpyrrolidin-1-yl)pentyloxy]quinolin-2(1H)-one formate salt***(QN18)**. Following General Procedure B, the reaction of a mixture of potassium carbonate (345.5 mg, 2.5 mmol), 7-hydroxyquinolin-2(1*H*)-one (80.6 mg, 0.5 mmol), and 1,5-dibromopentane (230 mg, 1.0 mmol) in acetonitrile (5 mL) afforded a crude intermediate that was suspended in acetonitrile (5 mL) and treated with (*S*)-2-methylpyrrolidine (170.3 mg, 2.0 mmol) to give compound **QN18** as free base. Treatment with 0.1% formic acid/H_2_O afforded compound **QN18** (27.01 mg, 10% yield). ^1^H-NMR (400 MHz, DMSO-*d*_6_) δ: 11.50 (br s, 1H), 7.80 (d, *J* = 9.5 Hz, 1H), 7.55 (dd, *J* = 8.3, 1,2 Hz 1H), 6.80–6.77 (m, 2H), 6.29 (d, *J* = 9.5 Hz, 1H), 4.00 (t, *J* = 6.5 Hz, 2H), 3.05–2.98 (m, 1H), 2.75–2.65 (m, 1H), 2.26–2.12 (m, 1H), 2.03–1.94 (m, 2H), 1.87–1.74 (m, 3H), 1.64–1.58 (m, 2H), 1.51–1.40 (m, 4H), 1.29–1.25 (m, 1H), 1.00 (d, *J* = 6.0 Hz, 3H). LC/MS *m*/*z* 315.2 (M + H), t_R_ = 0.49 min.

*7-[(1-Methylpiperidin-4-yl)oxy]quinolin-2(1H)-one***(QN19)**. A mixture of 7-hydroxyquinolin-2(1*H*)-one (80.6 mg, 0.5 mmol), 1-methylpiperidin-4-ol (172.8 mg, 1.5 mmol), and cyanomethylenetributylphosphorane (362.0 mg, 1.5 mmol) in toluene (5 mL, 47.3 mmol) was heated at 100 °C for 16 h. The reaction mixture was diluted with methanol and loaded into 10 g SCX resin. Resin was washed with methanol, and crude product was eluted with 2N ammonia in methanol before evaporation of the solvent. The residue was purified by column chromatography (CH_2_Cl_2_/MeOH (from 1% to 5%)) to give compound **QN19** (46.1 mg, 30% yield). ^1^H-NMR (400 MHz, DMSO-*d*_6_) δ: 11.49 (s, 1H), 7.80 (d, *J* = 9.5 Hz, 1H), 7.55 (dd, *J*= 8.3, 1,3 Hz, 1H), 6.82–6.79 (m, 2H), 6.30 (d, *J* = 9.5 Hz, 1H), 4.38 (tt, *J* = 8.2, 4.0 Hz, 1H), 3.17 (d, *J* = 5.2 Hz, 1H), 2.62–2.58 (m, 2H), 2.19 (s, 3H), 2.16–2.12 (m, 1H), 1.98–1.95 (m, 2H), 1.71–1.62 (m, 2H). LC/MS *m*/*z* 259.2 (M + H), t_R_ = 0.67 min.

#### 3.1.3. General Procedures for the Synthesis of DihydroQuinolinones (DQNs)

(a) General Procedure A via Mitsunobu reaction: A mixture of 7-hydroxy-3,4-dihydroquinolin-2(1*H*)-one (0.5 mmol), the appropriate aminoalcohol (0.5 mmol), and cyanomethylenetributylphosphorane (0.7 mmol) in toluene (5 mL) was heated at 100 °C for 16 h. The reaction mixture was diluted with methanol and loaded into 10 g SCX resin. Resin was washed with methanol, and crude product was eluted in 2N ammonia in methanol before evaporation of the solvent. The residue was purified by column chromatography (CH_2_Cl_2_/MeOH (from 1% to 5%)).

(b) General Procedure B via *O*-Alkylation: Step 1. A mixture of potassium carbonate (2.5 mmol), 7-hydroxy-3,4-dihydroquinolin-2(1H)-one (0.5 mmol), and the corresponding 1,*n*-dibromoalkane (1.0 mmol) in acetonitrile (5 mL) was heated at 50 °C for 20 h. After cooling, acetonitrile (10 mL) was added, the inorganic salts were filtered off, and the solvent was evaporated. Step 2. To a stirred solution of the crude intermediate in acetonitrile (5 mL), we added the appropriate amine (2.0 mmol). After stirring at 50 °C for 15 h, we diluted the reaction mixture with methanol and loaded it into 10 g SCX resin. Resin was washed with methanol, and crude product was eluted with 2N ammonia in methanol before evaporation of the solvent. The residue was purified by column chromatography [CH_2_Cl_2_/MeOH (from 1% to 5%)].

(c) General Procedure C via *O*-Alkylation: Step 1. A mixture of potassium carbonate (2.5 mmol), 7-hydroxy-3,4-dihydroquinolin-2(1H)-one (0.5 mmol), and the corresponding 1,*n*-dibromoalkane (1.0 mmol) in acetonitrile (5 mL) was heated at 50 °C for 20 h. After cooling, acetonitrile (10 mL) was added, the inorganic salts were filtered off, and the solvent was evaporated. Step 2. To a stirred solution of the crude intermediate in acetonitrile (5 mL), we added the appropriate amine (0.5 mmol). After stirring at 50 °C for 15 h, we diluted the reaction mixture with methanol and loaded it into 10 g SCX resin. Resin was washed with methanol, and crude product was eluted with 2N ammonia in methanol before evaporation of the solvent. The residue was purified by column chromatography (CH_2_Cl_2_/MeOH (from 1% to 5%)).

*7-(2-Morpholinoethoxy)-3,4-dihydroquinolin-2(1H)-one* (**DQN1**). Following General Procedure A, 7-hydroxy-3,4-dihydroquinolin-2(1*H*)-one (81.6 mg, 0.5 mmol) and 2-morpholinoethan-1-ol (65.6 mg, 0.5 mmol) were reacted with cyanomethylenetributylphosphorane (169 mg, 0.7 mmol) in toluene (5 mL, 47.3 mmol) to give compound **DQN1** (110.47 mg, 80% yield). ^1^H-NMR (400 MHz, DMSO-*d*_6_) δ: 9.97 (s, 1H), 7.05 (d, *J* = 8.2 Hz, 1H), 6.50 (dd, *J* = 8.2, 2.6 Hz, 1H), 6.43 (d, *J* = 2.6 Hz, 1H), 4.00 (t, *J* = 5.7 Hz, 2H), 3.59–3.57 (m, 4H), 2.80–2.77 (m, 2H), 2.68–2.65 (m, 2H), 2.47–2.40 (m, 6H). LC/MS *m*/*z* 277.2 (M + H), t_R_ = 0.63 min.

*7-[2-(4-Methylpiperazin-1-yl)ethoxy]-3,4-dihydroquinolin-2(1H)-one* (**DQN2**). Following General Procedure A, 7-hydroxy-3,4-dihydroquinolin-2(1*H*)-one (81.6 mg, 0.5 mmol) and 2-(4-methylpiperazin-1-yl)ethan-1-ol (72.1 mg, 0.5 mmol) were reacted with cyanomethylenetributylphosphorane (169 mg, 0.7 mmol) in toluene (5 mL, 47.3 mmol) to give compound **DQN2** (119.38 mg, 80% yield). ^1^H-NMR (400 MHz, DMSO-*d*_6_) δ: 9.97 (s, 1H), 7.05 (d, *J* = 8.3 Hz, 1H), 6.50 (dd, *J* = 8.3, 2.6 Hz, 1H), 6.43 (d, *J* = 2.6 Hz, 1H), 3.99 (t, *J* = 5.8 Hz, 2H), 2.78 (t, *J* = 7.5 Hz, 2H), 2.65 (t, *J* = 5.8 Hz, 2H), 2.52–2.48 (m, 4H), 2.43–2.40 (m, 4H), 2.31–2.25 (m, 2H), 2.15 (s, 3H). LC/MS *m*/*z* 290.2 (M + H), t_R_ = 0.60 min.

*7-[4-(4-Methylpiperazin-1-yl)butoxy]-3,4-dihydroquinolin-2(1H)-one***(DQN3)**. Following General Procedure C, the reaction of a mixture of potassium carbonate (345.5 mg, 2.5 mmol), 7-hydroxy-3,4-dihydroquinolin-2(1*H*)-one (81.6 mg, 0.5 mmol), and 1,4-dibromobutane (216 mg, 1.0 mmol) in acetonitrile (5 mL) afforded a crude intermediate that was suspended in acetonitrile (5 mL), and treated with *N*-methylpiperazine (50.08 mg, 0.5 mmol) to give compound **DQN3** (41.70 mg, 30% yield). ^1^H-NMR (400 MHz, DMSO-*d*_6_) δ: 9.97 (s, 1H), 7.04 (d, *J* = 8.2 Hz, 1H), 6.48 (dd, *J* = 8.3, 2.6 Hz, 1H), 6.43 (d, *J* = 2.6 Hz, 1H), 3.90 (t, *J* = 6.4 Hz, 2H), 2.78 (dd, *J* = 8.5, 6.5 Hz, 2H), 2.43–2.39 (m, 4H), 2.34–2.24 (m, 8H), 2.14 (s, 3H), 1.75–1.63 (m, 2H), 1.59–1.47 (m, 2H). LC/MS *m*/*z* 318.2 (M + H), t_R_ = 0.72 min.

*7-[5-(4-Methylpiperazin-1-yl)pentyloxy]-3,4-dihydroquinolin-2(1H)-one***(DQN4)**. Following General Procedure C, the reaction of a mixture of potassium carbonate (345.5 mg, 2.5 mmol), 7-hydroxy-3,4-dihydroquinolin-2(1*H*)-one (81.6 mg, 0.5 mmol), and 1,5-dibromopentane (230 mg, 1.0 mmol) in acetonitrile (5 mL) afforded a crude intermediate that was suspended in acetonitrile (5 mL), and treated with *N*-methylpiperazine (50.08 mg, 0.5 mmol) to give compound **DQN4** (43.22 mg, 30% yield). ^1^H-NMR (400 MHz, DMSO-*d*_6_) δ: 9.97 (s, 1H), 7.04 (d, *J* = 8.2 Hz, 1H), 6.48 (dd, *J* = 8.3, 2.6 Hz, 1H), 6.43 (d, *J* = 2.6 Hz, 1H), 3.88 (t, *J* = 6.5 Hz, 2H), 2.80–2.76 (m, 2H), 2.43–2.39 (m, 5H), 2.38–2.19 (m, 7H), 2.14 (s, 3H), 1.69–1.65 (m, 2H), 1.52–1.31 (m, 4H). LC/MS *m*/*z* 332.2 (M + H), t_R_ = 0.79 min.

*7-[2-(4-Isopropylpiperazin-1-yl)ethoxy]-3,4-dihydroquinolin-2(1H)-one***(DQN5)**. Following General Procedure A, 7-hydroxy-3,4-dihydroquinolin-2(1*H*)-one (81.6 mg, 0.5 mmol) and 2-(4-isopropylpiperazin-1-yl)ethan-1-ol (63.6 mg, 0.5 mmol) were reacted with cyanomethylenetributylphosphorane (169 mg, 0.7 mmol) in toluene (5 mL) to give compound **DQN5** (46.62 mg, 30% yield). ^1^H-NMR (400 MHz, DMSO-*d*_6_) δ: 9.97 (s, 1H), 7.05 (d, *J* = 8.3 Hz, 1H), 6.50 (dd, *J* = 8.3, 2.6 Hz, 1H), 6.43 (d, *J* = 2.6 Hz, 1H), 3.98 (t, *J* = 5.8 Hz, 2H), 2.78 (t, *J* = 7.5 Hz, 2H), 2.65–2.59 (m, 4H), 2.47–2.37 (m, 9H), 0.96 (d, *J* = 6.5 Hz, 6H). LC/MS *m*/*z* 318.2 (M + H), t_R_ = 0.72 min.

*7-[3-(4-Isopropylpiperazin-1-yl)propoxy]-3,4-dihydroquinolin-2(1H)-one***(DQN6)**. Following General Procedure C, the reaction of a mixture of potassium carbonate (345.5 mg, 2.5 mmol), 7-hydroxy-3,4-dihydroquinolin-2(1*H*)-one (81.6 mg, 0.5 mmol), and 1,3-dibromopropane (202 mg, 1.0 mmol) in acetonitrile (5 mL) afforded a crude intermediate that was suspended in acetonitrile (5 mL), and treated with 1-isopropylpiperazine (64.1 mg, 0.5 mmol) to give compound **DQN6** (31.79 mg, 20% yield). ^1^H-NMR (400 MHz, DMSO-*d*_6_) δ: 9.96 (br s, 1H), 7.04 (d, *J* = 8.2 Hz, 1H), 6.48 (dd, *J* = 8.2, 2.6 Hz, 1H), 6.43 (d, *J* = 2.6 Hz, 1H), 3.91 (t, *J* = 6.4 Hz, 2H), 2.78 (dd, *J* = 8.5, 6.5 Hz, 2H), 2.62–2.55 (m, 2H), 2.49–2.40 (m, 6H), 2.40–2.31 (m, 5H), 1.86–1.80 (m, 2H), 0.96 (d, *J* = 6.5 Hz, 6H). LC/MS *m*/*z* 332.2 (M + H), t_R_ = 0.78 min.

*7-[4-(4-Isopropylpiperazin-1-yl)butoxy]-3,4-dihydroquinolin-2(1H)-one***(DQN7)**. Following General Procedure C, the reaction of a mixture of potassium carbonate (345.5 mg, 2.5 mmol), 7-hydroxy-3,4-dihydroquinolin-2(1*H*)-one (81.6 mg, 0.5 mmol), and 1,4-dibromobutane (216 mg, 1.0 mmol) in acetonitrile (5 mL) afforded a crude intermediate that was suspended in acetonitrile (5 mL), and treated with 1-isopropylpiperazine (64.1 mg, 0.5 mmol) to give compound **DQN7** (58.01 mg, 30% yield). ^1^H-NMR (400 MHz, DMSO-*d*_6_) δ: 9.97 (s, 1H), 7.04 (d, *J* = 8.2 Hz, 1H), 6.48 (dd, *J* = 8.2, 2.6 Hz, 1H), 6.43 (d, *J* = 2.6 Hz, 1H), 3.90 (t, *J* = 6.4 Hz, 2H), 2.78 (t, *J* = 7.5 Hz, 2H), 2.59–2.54 (m, 3H), 2.46–2.37 (m, 6H), 2.36–2.23 (m, 4H), 1.71–1.65 (m, 2H), 1.55–1.51 (m, 2H), 0.95 (d, *J* = 6.5 Hz, 6H). LC/MS *m*/*z* 346.2 (M + H), t_R_ = 0.83 min.

*7-[5-(4-Isopropylpiperazin-1-yl]pentyloxy)-3,4-dihydroquinolin-2(1H)-one***(DQN8)**. Following General Procedure C, the reaction of a mixture of potassium carbonate (345.5 mg, 2.5 mmol), 7-hydroxy-3,4-dihydroquinolin-2(1*H*)-one (81.6 mg, 0.5 mmol), and 1,4-dibromopentane (230 mg, 1.0 mmol) in acetonitrile (5 mL) afforded a crude intermediate that was suspended in acetonitrile (5 mL), and treated with 1-isopropylpiperazine (64.1 mg, 0.5 mmol) to give compound **DQN8** (50.70 mg, 30% yield). ^1^H-NMR (400 MHz, DMSO-*d*_6_) δ: 9.97 (s, 1H), 7.04 (d, *J* = 8.2 Hz, 1H), 6.48 (dd, *J* = 8.2, 2.6 Hz, 1H), 6.43 (d, *J* = 2.6 Hz, 1H), 3.88 (t, *J* = 6.5 Hz, 2H), 2.78 (t, *J* = 7.5 Hz, 2H), 2.60–2.54 (m, 4H), 2.47–2.40 (m, 5H), 2.34–2.32 (m, 2H), 2.26–2.22 (m, 2H), 1.73–1.66 (m, 2H), 1.50–1.34 (m, 4H), 0.95 (d, *J* = 6.5 Hz, 6H). LC/MS *m*/*z* 360.2 (M + H), t_R_ = 0.90 min.

*7-[2-(Diethylamino)ethoxy]-3,4-dihydroquinolin-2(1H)-one***(DQN9)**. Following General Procedure A, 7-hydroxy-3,4-dihydroquinolin-2(1*H*)-one (81.6 mg, 0.5 mmol) and 2-(diethylamino)ethan-1-ol (65.6 mg, 0.5 mmol) were reacted with cyanomethylenetributylphosphorane (169 mg, 0.7 mmol) in toluene (5 mL, 47.3 mmol) to give compound **DQN9** (97.32 mg, 70% yield). ^1^H-NMR (400 MHz, DMSO-*d*_6_) δ: 9.96 (s, 1H), 7.04 (d, *J* = 8.2 Hz, 1H), 6.48 (dd, *J* = 8.2, 2.6 Hz, 1H), 6.43 (d, *J* = 2.6 Hz, 1H), 3.93 (t, *J* = 6.2 Hz, 2H), 2.76 (q, *J* = 7.1 Hz, 4H), 2.59–2.50 (m, 4H), 2.42 (t, *J* = 6.2 Hz, 2H), 0.97 (t, *J* = 7.1 Hz, 6H). LC/MS *m*/*z* 263.2 (M + H), t_R_ = 0.82 min.

*7-[3-(Diethylamino)propoxy]-3,4-dihydroquinolin-2(1H)-one***(DQN10)**. Following General Procedure B, the reaction of a mixture of potassium carbonate (345.5 mg, 2.5 mmol), 7-hydroxy-3,4-dihydroquinolin-2(1*H*)-one (81.6 mg, 0.5 mmol), and 1,3-dibromopropane (202 mg, 1.0 mmol) in acetonitrile (5 mL) afforded a crude intermediate that was suspended in acetonitrile (5 mL), and treated with diethylamine (146.28 mg, 2.0 mmol) to give compound **DQN10** (63.15 mg, 50% yield). ^1^H-NMR (400 MHz, DMSO-*d*_6_) δ: 9.96 (s, 1H), 7.04 (d, *J* = 8.2 Hz, 1H), 6.48 (dd, *J* = 8.2, 2.6 Hz, 1H), 6.43 (d, *J* = 2.6 Hz, 1H), 3.92 (t, *J* = 6.3 Hz, 2H), 2.78 (dd, *J* = 8.4, 6.5 Hz, 2H), 2.50–2.37 (m, 8H), 1.81–1.74 (m, 2H), 0.94 (t, *J* = 7.1 Hz, 6H). LC/MS *m*/*z* 277.2 (M + H), t_R_ = 0.84 min.

*7-[4-(Diethylamino)butoxy]-3,4-dihydroquinolin-2(1H)-one***(DQN11)**. Following General Procedure B, the reaction of a mixture of potassium carbonate (345.5 mg, 2.5 mmol), 7-hydroxy-3,4-dihydroquinolin-2(1*H*)-one (81.6 mg, 0.5 mmol), and 1,3-dibromobutane (216 mg, 1.0 mmol) in acetonitrile (5 mL) afforded a crude intermediate that was suspended in acetonitrile (5 mL), and treated with diethylamine (146.28 mg, 2.0 mmol) to give compound **DQN11** (80.66 mg, 60% yield). ^1^H-NMR (400 MHz, DMSO-*d*_6_) δ: 9.97 (s, 1H), 7.04 (d, *J* = 8.2 Hz, 1H), 6.48 (dd, *J* = 8.2, 2.6 Hz, 1H), 6.43 (d, *J* = 2.6 Hz, 1H), 3.90 (t, *J* = 6.5 Hz, 2H), 2.78 (t, *J* = 7.5 Hz, 2H), 2.47–2.37 (m, 8H), 1.72–1.65 (m, 2H), 1.53–1.46 (m, 2H), 0.95 (t, *J* = 7.1 Hz, 6H). LC/MS *m*/*z* 291.2 (M + H), t_R_ = 0.87 min.

*7-[5-(Diethylamino)pentyloxy]-3,4-dihydroquinolin-2(1H)-one***(DQN12)**. Following General Procedure B, the reaction of a mixture of potassium carbonate (345.5 mg, 2.5 mmol), 7-hydroxy-3,4-dihydroquinolin-2(1*H*)-one (81.6 mg, 0.5 mmol), and 1,3-dibromopentane (230 mg, 1.0 mmol) in acetonitrile (5 mL) afforded a crude intermediate that was suspended in acetonitrile (5 mL) and treated with diethylamine (146.28 mg, 2.0 mmol) to give compound **DQN12** (76.63 mg, 50% yield). ^1^H-NMR (400 MHz, DMSO-*d*_6_) δ: 9.97 (s, 1H), 7.04 (d, *J* = 8.2 Hz, 1H), 6.48 (dd, *J* = 8.2, 2.6 Hz, 1H), 6.43 (d, *J* = 2.6 Hz, 1H), 3.88 (t, *J* = 6.5 Hz, 2H), 2.78 (t, *J* = 7.5 Hz, 2H), 2.48–2.37 (m, 6H), 2.35 (t, *J* = 6.6 Hz, 2H), 1.73–1.66 (m, 2H), 1.44–1.38 (m, 4H), 0.94 (t, *J* = 7.1 Hz, 6H). LC/MS *m*/*z* 305.2 (M + H), t_R_ = 0.95 min.

*7-[(1-Methylpiperidin-4-yl)oxy]-3,4-dihydroquinolin-2(1H)-one***(DQN13)**. A mixture of 7-hydroxy-3,4-dihydroquinolin-2(1*H*)-one (81.6 mg, 0.5 mmol), 1-methylpiperidin-4-ol (172.8 mg, 1.5 mmol), and cyanomethylenetributylphosphorane (362.0 mg, 1.5 mmol) in toluene (5 mL, 47.3 mmol) was heated at 100 °C for 16 h. The reaction mixture was diluted with methanol, loaded into 10 g SCX resin. Resin was washed with methanol, and crude product was eluted in 2N ammonia in methanol before evaporation of the solvent. The residue was purified by column chromatography (CH_2_Cl_2_/MeOH (from 1% to 5%)) to give compound **DQN13** (87.79 mg, 70% yield). ^1^H-NMR (400 MHz, DMSO-*d*_6_) δ: 9.93 (s, 1H), 7.04 (d, *J* = 8.3 Hz, 1H), 6.50 (dd, *J* = 8.3, 2.6 Hz, 1H), 6.44 (d, *J* = 2.6 Hz, 1H), 4.24-4.21 (m, 1H), 2.78 (t, *J* = 7.5 Hz, 2H), 2.61–2.57 (m, 2H), 2.46–2.40 (m, 2H), 2.20 (s, 3H), 2.17–2.09 (m, 2H), 1.92–1.87 (m, 2H), 1.65–1.58 (m, 2H). LC/MS *m*/*z* 261.2 (M + H), t_R_ = 0.70 min.

### 3.2. Biological Evaluation

#### 3.2.1. Human MAO Activity Assay

The *hr*MAO-A and *hr*MAO-B enzymes were purchased from Sigma-Aldrich (St. Louis, MO, USA). The reaction mixture contained *hr*MAO-A (2.5 µg/mL protein final concentration) or *hr*MAO-B (6.25 µg/mL protein final concentration) enzyme and tested compound in final concentration of 1 and 10 µM in 50 mM potassium phosphate buffer with 20% (v/v) glycerol (pH 7.5). The mixture was pre-incubated at 37 °C for 5 min and subsequently substrate kynuramine was added to the final concentration of 60 µM in the case of *hr*MAO-A and 30 µM in the case of *hr*MAO-B. The final volume of reaction mixture was 0.1 mL. The whole reaction mixture was incubated at 37 °C for 30 min. The reaction was stopped by the addition of 200 µL acetonitrile/methanol mixture (ratio 1:1) and cooling down to 0 °C. The sample was then centrifuged (16,500× *g*) for 10 min. The deamination product of kynuramine formed during the enzymatic reaction 4-hydroxyquinoline (4-HQ) was determined by HPLC–MS on a 2.1 mm × 50 mm, 1.8 µm Zorbax RRHD Eclipse plus C18 column (Agilent) by using a 6470 Series Triple Quadrupole mass spectrometer (Agilent) (electrospray ionisation—positive ion mode). Three MRM transitions were followed for kynuramine (165.1 => 30.2, 165.1 => 118.0, 165.1 => 136.0) and 4-HQ (146.1 => 51.1, 146.1 => 77.0, 146.1 => 91.0). Eluents: (A) 0.1% formic acid in water; (B) 0.1% formic acid in acetonitrile.

#### 3.2.2. Human Esterase Activity Assay

##### Inhibition Efficiency Screening and Determination of IC_50_

The *hr*AChE and *hr*BuChE were prepared as recombinant proteins at the University of Hradec Kralove. For their production, the mammalian expression system was used. Briefly, the DNA sequence encoding human AChE and BuChE was obtained from UniProtKB Server (www.uniprot.org, accession numbers: P22303 and P06276) and de novo synthesized as GeneArt Strings DNA fragments by GeneArt Gene Synthesis Service (Thermo Fisher Scientific, Pardubice, Czech Republic). The DNA fragments were PCR-amplified using gene-specific primers, adding the DNA sequence for C-terminal 6× His-tag. The amplicons were inserted into the mammalian pcDNA3.4 vector by TOPO cloning technology, and the final DNA constructs were verified by Sanger sequencing (ABI PRISM 3130xl). For protein expression, the DNA constructs were transiently transfected into Hek293 derivatives. Recombinant proteins were collected from culture supernatant 6 days later and stored at −80 °C for further purification.

The *hr*AChE and *hr*BuChE were purified using a NGC Medium-Pressure Chromatography System (Bio-Rad, USA) [25]. The total volume of 6–8 mL of medium containing secreted protein was desalted using 5 mL HiTrap Desalting column (GE Healthcare, Prague, Czech Republic) equilibrated with buffer A (20 mM sodium phosphate buffer, 150 mM NaCl, 15 mM imidazole, and 20% glycerol; pH 7.4). Acquired supernatant was loaded onto a 1 mL HisTrap FF column (GE Healthcare, Prague, Czech Republic) equilibrated with buffer A. Captured proteins were eluted with buffer B (20 mM sodium phosphate buffer, 150 mM NaCl, 500 mM imidazole, and 20% glycerol; pH 7.4). Imidazole was subsequently removed by repeated centrifugation in Amicon Ultra-4 (Ultracel-10K) tube (Merck Millipore). Protein concentration was determined by linearized Bradford method adapted for 96-well plate.

The catalytic activity of enzymes was determined by standard Ellman method [26] adapted for 96-well plates. The reaction mixture contained *hr*AChE (70 ng/mL protein final concentration) or *hr*BuChE (220 ng/mL protein), tested compound at required concentration (varying from 0.1 µM up to 80 µM), and 500 µM 5,5′-dithiobis-2-nitrobenzoic acid (DTNB) in 20 mM sodium phosphate buffer (pH 7.4). The mixture was pre-incubated at 37 °C for 15 min, and subsequently substrate acetylthiocholine iodide (ATChI) or butyrylthiocholine iodide (BuTChI) was added to the final concentration of 1000 µM. The final volume of reaction was 100 µL. The product formed during the reaction 5-thio-2-nitrobenzoic acid (TNB) was determined by following its absorbance at 436 nm. The catalytic activity was evaluated as amount of product (%) formed by enzyme after 10 min of incubation at 37 °C. IC_50_ values of individual compounds were determined by non-linear regression using GraphPad Prism 7.

#### 3.2.3. Determination of Inhibition Kinetics

Compound with the highest inhibition potential against *hr*AChE was further analysed regarding its inhibition kinetics parameters (inhibition mechanism and inhibitory constant). Thus, esterase activity assay was carried out at various concentrations of substrate ATChI (ranging from 25 µM to 2000 µM) and various concentrations of tested compound (0.1 µM, 0.3 µM, and 1 µM). Inhibition mechanism and kinetic constant were determined by non-linear regression and double reciprocal method by Lineweaver-Burk [27] using GraphPad Prism 7.

### 3.3. Docking Analysis

Compound **QN8** was prepared using “build” option within Discovery Studio 2.1 to create three-dimensional geometry, and assign proper bond orders and ionization states prior to virtual screening. Protein crystal structure of *h*AChE (PDB ID: 1B41) was prepared prior to docking, using protein model tool in Discovery Studio in order to add hydrogen atoms and to assign proper bonds, bond orders, hybridization, and charges. In addition, cocrystal ligands and water molecules were removed. AutoDockTools (ADT; version 1.5.4) was used to add hydrogens and partial charges for proteins and ligands using Gasteiger charges. To give flexibility to the binding site, residues lining the AChE (Trp286, Tyr124, Tyr337, Tyr341, Tyr72, Asp74, Thr75, Trp86) were allowed to move during the docking search as performed by software AutoDock Vina 1.1.2 [29]. The ADT program was used to generate the docking input files. Docking calculations were performed with the program AutoDock Vina 1.1.2 and default parameters were used except num modes, which was set to 40. The grid box was built with a resolution of 1 Å and 60 × 60 × 72 points, and it was positioned at the middle of the protein (*x* = 116.546; *y* = 110.33; *z* = −134.181). The best Vina scored poses were considered and the docked ligand output files were viewed and analysed using Discovery Studio.

## 4. Conclusions

In this work, we described the synthesis of 19 quinolinones (**QN1**-**19**) and 13 dihydroquinolinones (**DQN1**-**13**) designed as potential MSM for AD therapy. **QN** and **DQN** ligands were easily synthesized by Mitsunobu or *O*-alkylation protocols starting from 7-hydroxyquinolin-2(1*H*)-one (**1**) (Scheme 1) and 7-hydroxy-3,4-dihydroquinolin-2(1*H*)-one (**2**) (Scheme 2), respectively, and suitably functionalized precursors.

The biological analysis of these compounds on selected targets (*hr*ChEs/*hr*MAOs) involved in the pathology of the disease produced interesting results. Thus, contrary to our expectations, none of them showed significant *hr*MAO inhibition. However, molecules **QN8**, **QN9,** and **DQN7** showed promising *hr*AChE and *hr*BuChE inhibition. In particular, molecule **QN8** resulted as a potent and selective non-competitive *hr*AChE inhibitor (IC_50_ = 0.29 µM), with K_i_ value in the nanomolar range (79 nM).

Pertinent docking analysis of hit-compound **QN8** confirmed the observed kinetic and *hr*ChE inhibition results, suggesting that this ligand is an interesting hit for further investigation. In addition, theoretical physico-chemical property analysis of **QN8** confirmed that this is an attractive ligand for deeper investigation in the search of more efficient molecules for AD therapy.

## Figures and Tables

**Table 1:** Determination of IC_50_ for *human recombinant* acetylcholinesterase (*hr*AChE) and butyrylcholinesterase (*hr*BuChE) inhibition by ligands **QN8**, **QN9**, **DQN7,** and donepezil.

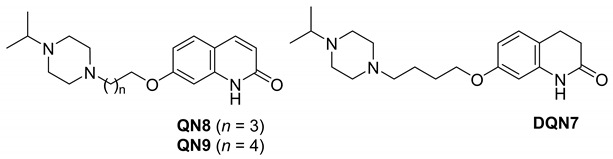			
Compound	IC_50_ (µM) ^a^		Selectivity Index IC_50_ (*hr*BuChE)/IC_50_ (*hr*AChE)
	*hr*AChE	*hr*BuChE	
**QN8**	0.29 ± 0.02	12.73 ± 0.45	44
**QN9**	0.96 ± 0.04	6.70 ± 0.32	7
**DQN7**	1.58 ± 0.15	16.73 ± 0.46	11
**Donepezil [28]**	0.048 ± 0.003	3.17 ± 0.1	66

^a^ IC_50_ values are expressed as the mean ± standard error of the mean for triplicate determinations.

**Table 2:** Physico-chemical properties of compound **QN8** calculated using QikProp. ^a.^

**Molecule**	**MW**	**SASA**	**Volume**	**DonorHB**	**AccptHB**	**QPlogPo/w**	**QPlogS**	
**QN8**	343.468	708.219	1221.055	1.000	7.250	2.449	−1.920	
**Molecule**	**QPPCaco**	**PSA**	**QPlogBB**	**metab**	**QPlogKhsa**	**% HOA**	**ROF**	**ROT**
**QN8**	87.998	60.557	−0.038	3	0.185	76.087	0	0

^a^ MW: molecular weight of the molecule (130–725). SASA: total solvent accessible surface area, in square angstroms, using a probe with a 1.4 Å radius (limits 300–1000). Volume: total solvent-accessible volume, in cubic angstroms, using a probe with a 1.4 Å radius (limits 500–2000). donorHB: estimated number of hydrogen bonds that would be accepted by the solute (limits: 0–6). accptHB: estimated number of hydrogen bonds that would be donated by the solute (limits: 2–20). QPlogPo/w: predicted octanol/water partition coefficient (limits −2.0–6.5). QPlogS: predicted aqueous solubility. S, in mol/dm^3^, is the concentration of the solute’s saturated solution that is in equilibrium with crystalline solid (limits −6.5–0.5). QPPCaco: predicted apparent Caco-2 cell permeability in nanometres per second. Caco-2 cells is a model for the gut–blood barrier. QikProp predictions are for non-active transport. (<25 poor, >500 great). PSA: Van der Waals surface area of polar nitrogen and oxygen atoms (limits 7–200). QPlog BB: predicted brain/blood partition coefficient (limits −3.0–1.2). metab: number of likely metabolic reactions (limits 1–8). QPlogKhsa: prediction of binding to human serum albumin (limits −1.5–1.5). HOA: predicted qualitative human oral absorption on 0% to 100% scale. ROF: number of violations of Lipinski’s Rule Of Five [30] (molecular weight <500, QPlogPo/w <5, number of hydrogen bond donor ≤5, number of hydrogen bond acceptors HB ≤10). ROT: number of violations of Jorgensen’s rule of three [31,32] (QPlogS>−5.7, QPCaco >22 nm/s, number of primary metabolites <7).
